# Understanding the problems developing a healthy living programme in patients with serious mental illness: a qualitative study

**DOI:** 10.1186/1471-244X-14-38

**Published:** 2014-02-14

**Authors:** Robert Pearsall, Susan Hughes, John Geddes, Anthony Pelosi

**Affiliations:** 1 Department of Psychiatry, Monklands Hospital, Airdrie, UK; 2 Community Health Clinic, Carluke, UK; 3 Department of Psychiatry, University of Oxford, Oxford, UK; 4 Regional Eating Disorders Unit, St John’s Hospital, Livingston, UK

**Keywords:** Healthy living, Physical health, Qualitative study, Serious mental illness, Healthy living programme

## Abstract

**Background:**

People with serious mental illness are at an increased risk of physical ill health. Mortality rates are around twice those of the general population with higher levels of cardiovascular disease, metabolic disease, diabetes, and respiratory illness. Although genetics may have a role in the physical health problems of these patients, lifestyle and environmental factors such as smoking, obesity, poor diet, and low levels of physical activity play a prominent part.

**Methods:**

A qualitative grounded theory approach was used to understand the problems experienced by these individuals when asked to attend a healthy living programme. Three main areas were explored: the influence of potential barriers, health problems, and general attitudes towards healthy living.

**Results:**

Thirteen patients were interviewed during the study. Many did not recall receiving an initial invitation letter to the programme. Several believed that there was no necessity to attend as they had already had recent routine health checks by their general practitioner. The patients’ current level of mental and physical health was important with symptoms such as depression, anxiety or arthritis affecting interest in the programme. Patients described that they found smoking enjoyable or calming in its effect. Dietary intake was determined by taste or gaining pleasure in eating certain types of food. Several lessons were learnt during this research that may aid future research and practice. Participation seemed to be better if the approach was first made by the patient’s own community keyworker. This contact may have provided a greater opportunity to explain the purpose and importance of the programme. Alternative appointments should be considered when certain patients are in better physical and mental health. Healthy living programmes need to be flexible and adaptive to individual patient needs. Assistance from their community worker may help engagement. Simple measures may improve participation and reduce potential barriers.

**Conclusion:**

These findings highlighted some of the problems encountered by patients when attempting to participate in a healthy living programme. These results may be useful when implementing future healthy living interventions for patients with serious mental disorders.

## Background

The physical health problems of individuals with serious mental illness are of increasing concern [1]. Physical disease in conditions such as schizophrenia tend to be under-detected and under-diagnosed [2]. Mortality rates are around twice that of the general population [3,4]. Higher levels of cardiovascular disease [5,6], metabolic disease [7], diabetes [8,9], and respiratory illness [10] have been found. Genetics may have a role [11] but lifestyle and environmental factors such as smoking, obesity, poor dietary intake, and low levels of physical activity certainly play a part.

There is a greater focus on patients’ physical health by mental health services [12]. However levels of cardiovascular risk factors remain high. Rates of smoking of up to 70% have been found in patients with schizophrenia [6] compared with about 25% in the general population [13]. Individuals with serious mental illness have a poorer diet than the non-mentally ill population [14]. The prevalence of obesity ranges from 40-60%, up to four times that of the non-mentally ill population [15,16] and levels of physical activity remain consistently low [17].

These patients have less access to medical care, poorer quality of care, and preventative health checks are less commonly completed in both primary and secondary care than in the general population [2,12,18]. The nature of the mental illness may also affect motivation to change their lifestyle [19]. Programmes to address risk factors such as smoking or obesity have had only limited impact [20,21]. Interventions made available by mainstream campaigns do not seem to address the health needs of people with serious mental illness [22].

This study explored the problems of developing a healthy living programme in individuals with serious mental illness. It proposed to understand the influence of potential barriers, health problems, and general attitudes towards healthy living in this population.

## Methods

### Design of study

A qualitative study using grounded theory was used to examine the attitudes, views, and experiences of patients who had declined to participate in a healthy living programme. Ethical approval was obtained from Lanarkshire Local Research Ethics Committee.

### Participants

Patients were recruited from individuals who had previously declined to take part in a healthy living programme based in a community mental health team [23]. The proposed intervention consisted of a 24 week programme of smoking cessation, dietary advice, weight reduction, and exercise. Eighty-four individuals with a diagnosis of schizophrenia, schizoaffective or bipolar affective disorder on the caseload of the community mental health team were invited to participate. Uptake was disappointing with only thirty patients agreeing to take part. Participants for the qualitative study were recruited using purposive sampling from the remaining 54 individuals. The criteria used for the sampling were age of participants (younger or older than 40 years), and male or female gender.

### Procedure

Patients were contacted by their own community keyworker to determine whether they would be interested in speaking to the researcher. Efforts were made to reduce potential barriers such as problems with travel. Interviews were arranged at the patient’s home, if they were unable to attend the community base or community staff members were able to assist with transportation. Interviews were conducted by one member of the research team (RP). Written consent and permission to audio-tape the interview were obtained. Interviews were conducted until saturation of data occurred, at a point when no additional information or themes were generated.

Differing hypotheses had been proposed while attempting to understand the reasons for the problems encountered in developing the healthy living programme. Four main areas were explored. Firstly, the study sought to identify whether there were specific reasons such as travel difficulties, mental or physical health symptoms, or lack of interest. Secondly, attitudes towards healthy living were explored to determine, for example, whether individuals understood the risks of smoking or an unhealthy diet. Thirdly, difficulties experienced while attempting to stop smoking or change their diet were studied. Lastly, this research attempted to understand whether the concept of research or aspects of trial design discouraged individuals from taking part in the study.

### Interview

The main interview schedule had three main sections: attitudes towards attending a healthy living group; attitudes towards improving healthy living; and attitudes towards research. In each of these sections general topics were approached by direct questioning and a series of prompts were used to explore the respondents’ thinking on these issues. Supplementary questions were included for non-smokers or respondents who did not take any physical exercise.

All interviews were tape-recorded and notes were also taken during the interview, as required, if additional information was provided or observed. Feedback was also provided from community mental health team keyworkers following the interview and additional information recorded.

### Data analysis

Thematic analysis was used to analyse the data from the interviews. The data were initially openly coded (substantive coding) to form categories of information about the events or happenings [24]. Computer software [25] was used in the process of analysis. After open coding the data were assembled into larger axial codes to identify central or key phenomenon, exploring causal conditions, and actions or interactions that resulted from the central phenomenon. The process of analysis concluded with selective coding, integrating categories found during the axial coding stage.

## Results

Thirteen patients were interviewed over a 6 month period. Interviews lasted approximately 30-40 minutes. The mean age was 54.6 years; half were female.

### Results of interviews – specific themes

Thirteen interviews were analysed using process coding to understand the relevant concepts and categories from the respondents. From close analysis of the transcripts the following main themes were identified: (1) attitudes and experiences of patients towards a healthy living programme, (2) awareness of the risks of an unhealthy lifestyle, (3) knowledge of healthy living recommendations, (4) difficulty maintaining a healthy lifestyle, (5) readiness to change, (6) effect of research on participation, and (7) general themes. During the analysis some overlap of themes was observed, and some quotations may have been categorized into more than one theme.

### Attitudes and experiences of patients towards a healthy living programme

The initial part of the interview considered reasons for the patients’ reluctance to participate in the programme. Nearly half of respondents said they did not recall receiving a letter about the group, or that they had had mail problems at the time of recruitment. Issues such as problems opening mail, or a dislike of official type mail were not raised as a particular difficulty by respondents. At the time of the distribution of letters about the groups, corresponding copies of appointments were also sent to the patient’s keyworker in the community mental health team. The community keyworkers were asked to remind the patients to attend the intervention.

Patients in the remaining interviews gave a variety of explanations for their non-response to the invitational letters. Some indicated that they were aware of the invitation letter to the healthy living group, but said that they had had a recent health check and did not feel they needed to attend. One patient said:

“I have been well, yes, for five years. I have been well mentally and physically. I have good control over my diabetes; they are not worried about me at all with my diabetes; I get a six months check-up and then I get what they call a “MOT” they are calling it now doctor, they are calling it your MOT and I get everything tested and then I get the results in from my own GP and mostly he writes on it “excellent” – you know he puts like a wee comment in – it has either had “no comment” or “excellent” written on it – the cholesterol etc”.

The provision of health monitoring in primary care services seemed to reinforce a belief in some patients that they were in good health, and did not require to address risk factors (e.g smoking) and healthy living. It may be concluded therefore that information given at the start of a healthy living programme may need to indicate more clearly the purpose of the proposed intervention.

Another patient noted the following:

“I think at that time I had had my bloods checked by my Doctor quite recently.

My Doctor does a yearly review of my bloods - I am actually attending him at the moment for blood pressure”.

Some patients were unconcerned about their physical health. One patient said:

“To be honest with you, doctor, I am not being blunt because I am not a cheeky person, but I am being honest I don’t care about my physical health; I never have and I don’t think I ever will. 

It doesn’t worry me in the slightest – that is funny that. I take my medication because I know that would be silly not to but I don’t care how long I live and I don’t care what kills but I would like it to be in a hospital bed”.

Some patients displayed an indifference to their future and longevity of life. There was a sense of waiting to see how their life would go, with little urgency to address these current issues. Another patient commented during a discussion about life expectancy that they would just have to wait and see what happened in life (Table 1, quotation [a]).

**Table 1:** Problems developing a healthy living programme

**Themes and definitions**	**Representative transcript excerpts**
**Attitudes and experiences of patients towards a healthy living programme**	
[a] Refers to an indifference to their future and longevity of life.	“Is it – oh goodness. Well I will just have to wait and see what happens, doctor”.
[b] Refers to the effect of their health (both physical and mental) affecting the likelihood of participation in a healthy living group.	“I was thinking about it before I came to see you and I thought I will just be honest. I have no got – I don’t have the attitude of mind...”
	“Well this true, I mean, I am lucky so far that physically I am haven’t suffered a lot with pain; I don’t know what the story would be like if I was in pain”.
	“It is just my arthritis that gets me down. Strong painkillers help my hands, but my back and my knees and feet are very bad”.
**Awareness of the risks of an unhealthy lifestyle**	
[c] Refers to awareness of unhealthy aspects of their lifestyle, but failure to lead to a change in behaviour.	“The fat”.
[d] Refers to awareness of individuals about risk from, for example, their dietary intake. Effect of their salt intake on their hypertension,	“I have tried the semi-skimmed and I cannot stand it. I mean it would be okay if I wasn’t taking cereal. I could take it in tea and coffee but I can’t take it in cereal. I have got some and I flung it out”.
	“Clogs your arteries. Anything you like isn’t good for you - that is the problem”.
	“Well as you say reduce the salt. I never thought about that raises the blood pressure I am glad you told me about that”.

During the course of the interviews other themes emerged explaining why patients did not attend the programme. Some indicated that their health (both physical and mental) affected the likelihood of participation in a healthy living group. One respondent described that the group meeting would have been too much for her at the time, but since then her health has improved and that she may be able to attend. She noted the following:

“It would probably have been too much for me but see now I’m awright as long as I take the tablets – see if a don’t take my medication”.

Another respondent, although initially indicating that she did not remember being asked to attend, later mentioned that her arthritis had been affecting her, worsening her depression and implied that this was a factor explaining her difficulties attending the intervention (Table 1, quotation [b]).

One patient also described that she felt claustrophobic if she had to sit in a meeting. She noted:

“I can’t sit – claustrophobic – it is too hot”.

### Awareness of the risks of an unhealthy lifestyle

Awareness of the potential consequences of an unhealthy lifestyle and level of risk seemed poorly appreciated by many of the patients, despite the presence of risk factors such as smoking and poor dietary intake. One patient said that he did not have a cooker in his house, and ate out in an Indian restaurant every night. He noted that this was his only meal in the day, he enjoyed having his food prepared, and particularly liked the social contact. However he did not fully appreciate the consequences of this dietary intake in terms of the consumption of a high level of calories and saturated fat.

Another patient when asked responded that she did not eat any fruit or vegetables, as she did not particularly like them. She noted:

“No, nothing, virtually nothing”.

Another respondent found it very difficult to switch to low fat milk, and persisted with high fat milk, despite knowing of its potential problems (Table 1, quotation[c]).

During the course of the interviews this theme continued to be developed indicating that patients may be aware of unhealthy aspects of their lifestyle, but this failed to lead to a change in behaviour. Many patients seemed to describe unhealthy behaviour but without any awareness of the potential consequences. They lacked an urgency to change. In some cases lack of knowledge or information may explain unhealthy behaviour. One patient, who had had a stroke the previous year, was unaware of the effects of smoking on her risk of a further stroke.

“Aye – I tried to cut down but it is really, really hard – you get into the habit with a cup of coffee and you have the cigarette- a cup of tea and a cigarette and a cup of coffee and a cigarette.

When asked if she believed that stopping smoking cigarettes may prevent a further stroke said:

“No – I don’t really think it does - no.”

However despite explaining the potential consequences of continued smoking to this patient, she still seemed reluctant to consider abstaining.

Similarly another patient had not understood the effect of dietary intake of salt on high blood pressure (Table 1, quotation [d]).

### Knowledge of healthy living recommendations

Were patients aware of recommendations about healthy living such as dietary intake, weight or exercise, or would further health promotion be required in this population? During the interviews most patients seemed to have a reasonable understanding and knowledge of aspects of healthy living, such as dietary intake and levels of exercise. Knowledge of recommendations of a healthy diet, were evident in the majority of patients. One said:

“Pastas, fresh salads, oven cooking – I do like chicken, fish and all that in the oven rather than frying it – I don’t use the fryer at all it is usually oven cooking; pastas, rice, salads, soups”.

Several patients seemed to be aware of national dietary recommendations of daily intake of fruit and vegetables [26]. One person, although aware of recommended levels, found these difficult to achieve:

“Well it is about up to 5, I don’t think I quite manage the 5 – that would be quite difficult”.

Difficulty in achieving recommended levels of healthy living was noted by another patient (Table 2, quotation [a]). Patients indicated that they found it difficult to meet the recommended guidelines. It should be noted that the general population in Scotland does not have a good record of meeting these targets. The Scottish Health Survey [27] showed that only 20% of men and 24% of women meet the recommended daily amount of five or more portions per day of fruit and vegetables.

**Table 2:** Problems developing a healthy living programme

**Themes and definitions**	**Representative transcript excerpts**
**Knowledge of healthy living recommendations**	
[a] Refers to awareness of recommended levels of healthy living such as dietary intake.	“Well not very good. I certainly eat fruit and vegetables every day but I couldn’t say that I eat the five a day, maybe two or three a day, but I haven’t managed to make the five a day. I certainly make a point of eating fruit every day besides the vegetables I am taking with my meals. But I take a lot of ready-made meals, you know”.
**Difficulties maintaining a healthy lifestyle**	
[b] Refers to the difficulties individuals experience trying to maintain a healthy lifestyle. The problems of unpleasant effects on their physical and mental health when stopping smoking.	“Aye – just diet and that cut out a lot of fatty foods and takeaway food and that – cut that out. The only other thing is the smoking I try to cut that down but it is hard – I have been smoking for years. I tried to come off them before but with the mood swings my wife put me back on them because of the moods and that”.
[c] Refers to the difficulties individuals experience trying to maintain a healthy lifestyle. They described that if they were feeling unwell, or feeling depressed or had paranoid symptoms, they would be less likely to go out and be active illness.	“Aye – but see when I am depressed I don’t bother going out I just – when I am going to my mum’s I just take a taxi there and a taxi back”.
	“Well see before I was hospitalised I was one for walking everywhere. I used to walk from xxxx Street into Hamilton and back and thought nothing of it”.
	“When I am no well (*mental health*) I just do a lot less, but when I am well enough I dae like walking”.

### Difficulties maintaining a healthy lifestyle

In the interviews it was important to find out why patients found difficulty in changing aspects of their lifestyle.

#### Smoking

Patients gave differing reasons for not being able to stop smoking, such as pleasure from smoking, the calming effect of cigarettes, and the problems of weight gain or mood swings, when patients tried to stop.

One patient said:

“The world would be a sad place if I did nae get a ciggy. I can do a lot of chores about the house and then you can think about sitting down and having a cigarette almost as a treat. I know you would hate to hear me saying that, as a doctor I understand, but to me it is a treat and to do myself out of treats it is such a short life to do yourself out of the things that make you feel happy regardless”.

“Yes. It is a pleasure I would nae want to dae without, you know. I did stop each time I was pregnant, that was the incentive. I mean I can do it so I don’t feel so bad about that actually because I think I could stop I just don’t want to. I enjoy it too much it is like in my routine of the day and you know it would”.

Several patients also described a calming effect of smoking, one commented:

“It calms you down.

Yeah.

Well I am worried that I might feel unwell if I stop - that is another reason that I dinnae want to stop”.

Some patients described unpleasant effects on their physical and mental health when they previously attempted to stop smoking (Table 2, quotation [b]). During the interview this patient indicated that he became more irritable, moody and difficult to live with after he stopped smoking.

Some indicated that weight gain was the reason they did not stop smoking. The following two patients noted:

“I knew if I went off it again I could do but see for 2 years I had these twisting grinding feelings in my stomach and I would never have started smoking again - but, I mean, I felt dreadful going from a size 10 to a size 20”.

“I know if I could stop 80 Capstan at once I can get off 10 with help.

I know that it is bad for my health and I would never have started again - I just couldn’t get shot of the 4 stone in weight”

#### Dietary intake

Patients’ responses indicated a number of reasons for difficulty in maintaining a healthy diet. The most common theme that emerged suggested that many people just did not like the taste of certain foods, such as fruit and vegetables or skimmed milk.

“The one I take is the blue one - I think the skimmed one is the green top.

Well it is semi-skimmed I tried then and I didn’t like it at all. It is alright in things like tea and coffee but taking with cereal it is terrible. There is no taste in it”.

The remainder of the patients expressed other reasons for their dietary intake. One patient said she only ate food she fancied, another explained the reasons for her poor diet were laziness, in her words, to prepare healthier foods. Another patient said that she bought ready packet foods for convenience.

“I think it is laziness really. As I say I used to do more - I must get back to cooking more. We have got a very good butcher where I live”.

One patient indicated that boredom was the reason she ate unhealthily, that she ate when she felt fed up. One interviewee said he did not care what he ate, that he did not think he would live beyond age 50-60 years of age. He indicated he would be content to live to that number of years and had decided just to eat what he liked.

#### Exercise

Explanations for low levels of exercise were not answered very clearly by most patients. However a general theme that emerged indicated that current level of mental and physical health was an important factor in determining level of exercise. Patients indicated that if they were feeling unwell, or feeling depressed or had paranoid symptoms, they would be less likely to go out and be active (Table 2, quotation [c]).

One respondent described specific mental health symptoms that stopped her going out.

“Aye – most times I am usually with somebody. The paranoia and that is still quite – it is no as bad as it wiz but it is still there”.

### Readiness to change

During the interviews it was important to understand whether patients were ready to change aspects of their lifestyle that could contribute to physical health problems. Some examples emerged indicating that although patients wanted to change their behaviour there was no sense of immediacy to do so. One patient indicated that in the near future she would attempt to stop smoking, but would delay until after Christmas.

“I am going to try again after Christmas and New Year. I am going to try the patches or something and try again. I have tried with them and different things and see if I can dae the patches this time”.

Another patient wanted to return to a better diet but seemed to be reluctant to consider change at this time

“I think it is laziness really. As I say I used to do more - I must get back to cooking more. We have got a very good butcher where I live”.

Several respondents, particularly female patients, gave a sense that weight was an important factor for them which they wanted to change. However there was little sense of urgency. One patient said:

“Just to keep my weight down, aye, try to keep on top of that”.

### Effect of research on participation

During the interviews we tried to gain a sense of whether participation in a research study affected the recruitment to the healthy living group. Would patients have preferred to attend if a clinical intervention had been proposed, rather than a research study?

In the qualitative study most respondents noted favourably that research would not affect their participation. One patient said:

“No, no it wouldn’t put me off”.

Patients seemed generally helpful and interested in participating in research studies. However some may not have fully understood the meaning of the concept. One participant was asked if they knew what is meant by a research study. They said:

“No. Is it an assessment?”

### General themes

In the course of analysis two central themes arose from the interviews, which helped to explain some of the problems that patients experienced when considering a healthy living programme.

A general sense became clear that patients tended to describe that they lived in the “here and now”, that they did not plan their future or consider important issues that may affect longevity. They described a sense of immediacy or “short-termism” where they dealt with matters in the present and very much did not plan for tomorrow or the future. They intimated that life at times was difficult, having to cope with their mental health symptoms, and this affected their ability to look ahead to the future.

A further general theme emerged that many patients were poorly focussed on their level of healthy living and were not strongly motivated to change. Although some explicitly indicated these views in their interviews, a general sense emerged through most of the patients that change at this time would be difficult. They seemed to be aware of health risks such as smoking or poor diet but were unable to address these at this time. Patients seemed to be in a stage of inertia with a limited ability to make moves to improve these aspects of their lives. Patients’ lives seemed focussed on the current level of health with limited consideration of the consequences of these risk factors, and their effect on longevity.

## Discussion

The aim of this study was to understand the problems that patients with serious mental illness may experience in participating in healthy living programmes. Several lessons were learnt that may aid future research and practice. Firstly, the initial contact with the patient by mailed letter achieved a poor response. Participation seemed to be better if the approach was first made by the patient’s own community keyworker. This contact may have provided a greater opportunity to explain the purpose and importance of the programme. Secondly, the individual’s current level of mental and physical health was important. Alternative appointments should be available for when the individual is in better health. Thirdly, healthy living programmes need to be flexible and adaptive to individual patient needs. Assistance from their community worker may help their engagement.

In this qualitative research we tried to understand why people with serious mental illness found it difficult to lead more healthy lives and engage in lifestyle programmes available in the community. Existing literature to date has mainly focused on the problems of addressing specific risk factors such as stopping smoking or becoming more physically active. For example, Lawn et al [22] found that smoking played an important role in the lives of individuals with serious mental illness. Smoking alleviated some effects of the stigma of mental illness by giving patients a sense of freedom in the presence of overwhelming powerlessness to predict or decide their own future. In a recent study Kerr et al [28] identified that if individuals stopped smoking their anxiety levels would increase, they would lose an important coping resource, they would have given up something they found pleasurable and, most importantly, their mental health would deteriorate. These views overlap with some of the themes that emerged with our subjects.

Several studies have examined the potential barriers to and benefits of physical activity for people with serious mental illness. McDevitt et al [29] found that symptoms of mental illness (e.g lack of energy or volition), medication, weight gain from medication, and safety concerns restricted the ability to be physically active. Several barriers to the uptake of physical activity were identified by Johnstone et al [30], such as limited experience of previous physical activity, reduced self esteem and confidence, and lack of structure or planning to their day. They also found that side effects of medication, particularly sedation, weight gain, tremor, and sweating emerged as the most prominent barriers to physical activity. Our interviews also elicited the importance of mental health symptoms such as low mood, or paranoid beliefs in preventing increased levels of exercise.

Why has it proved so difficult to improve the physical health of people with serious mental illness? Different methods may need to be considered to address their problems. Early focus and recognition of physical health problems is important. Greater training in physical health care and health promotion should be offered to mental health practitioners. Targeted programmes such as smoking cessation should be available by each mental health provider to reduce risk factors in this population [1]. A recent editorial [31] recommended that when patients are admitted to psychiatric hospital this could be a valuable opportunity to identify and address some physical health problems. Mental health services may need to consider systematic screening of individuals with serious mental illness to avoid some patients at risk remaining untreated [32]. There may be a role for a specialist mental health nurse within mental health teams to address these issues [32]. Clearer guidelines may be helpful to inform clinicians. Existing guidelines primarily focus on screening and monitoring of cardiometabolic risk factors [33]. Additional information should be offered to advise practitioners on the best available method to improve the physical health of people with serious mental illness.

There are strengths and limitations to the results we have presented. Valuable findings found were that the use of single mailed letters to patients reduced recruitment. Participation seemed to be better if the approach was first made by the patient’s own community keyworker. The patients’ current level of mental and physical health was important and affected participation. Many patients failed to fully understand the purpose of the programme. This study has a number of limitations that need to be acknowledged. Access to all patients who had not attended the healthy living programme was not possible. The remaining individuals would not participate or could not be contacted directly. Recruitment may have been affected by several factors. Initial contact was made by the patient’s community keyworker and not the researcher. Lack of direct contact with potential participants may have affected the participation of individuals. It is difficult to know the degree of explanation or discussion of the study given by the community keyworker. In addition two keyworkers seemed reluctant to participate in the recruitment process. Unfortunately one of these workers had access to several patients who had not attended the initial healthy living programme. It is possible that we missed important data obtainable only from the patients who did not participate.

## Conclusions

In conclusion this study explored the difficulties of improving the healthy living of individuals with serious mental illness. These findings are important in the implementation of future healthy living programmes in order to reduce the persistently high levels of mortality and morbidity in this population.

## Competing interests

RP, SH, and AP declared no competing interests. JG has received research funding from MRC, ESRC, NIHR, Stanley Medical Research Institute and has received donations of drugs supplies for trials from Sanofi-Aventis and GSK. He has acted as an expert witness for Dr Reddys.

## Authors’ contributions

RP developed and conducted the research, the analysis, and drafted the manuscript. SH, AP, and JG contributed to the study design and drafting the manuscript. All authors read and approved the final manuscript.

## Pre-publication history

The pre-publication history for this paper can be accessed here:

http://www.biomedcentral.com/1471-244X/14/38/prepub
